# Neuroprotective Potential of Peptide KSVSPKFLTG from *Magallana gigas* via Activating the ACE2/Ang-(1-7)/Mas Axis in SHSY-5Y Cells

**DOI:** 10.3390/antiox15080992

**Published:** 2026-08-10

**Authors:** Junlong Lai, Zhiwei Zheng, Jialong Gao, Zhihong Zheng, Haisheng Lin, Zhongqin Chen, Guoping Zhu, Wenhong Cao, Xiaoyu Xia

**Affiliations:** 1 College of Food Science and Technology, Guangdong Ocean University, Zhanjiang 524088, Chinazzh617@gdou.edu.cn (Z.Z.);; 2 Shenzhen Institute of Guangdong Ocean University, Shenzhen 518108, China; 3 National Research and Development Branch Center for Shellfish Processing (Zhanjiang), Guangdong Provincial Key Laboratory of Aquatic Products Processing and Safety, Guangdong Provincial Engineering Technology Research Center of Seafood, Zhanjiang 524088, China

**Keywords:** oyster peptide, ACE2, renin–angiotensin system, neuroprotection

## Abstract

Given the growing demand for health-promoting foods, there is increasing interest in the multi-dimensional regulatory effects and the mechanisms of food-derived bioactives. Here, we report that KSVSPKFLTG, an anti-inflammatory peptide from *Magallana gigas* with ACE2-binding properties, protects SHSY-5Y cells against MPP^+^/FAC-induced neurotoxicity. The peptide attenuates oxidative stress damage in SHSY-5Y cells and improves the mitochondrial membrane potential. It also mitigates iron overload and upregulates ferroptosis-related protective proteins (GPX4, SLC7A11, BCBP2, SLC40A1) via activating the renin–angiotensin system. These findings reveal an RAS-modulating neuroprotective mechanism of the oyster-derived anti-inflammatory peptide, simultaneously opening new avenues for alleviating nerve damage caused by inflammation through bioactive peptides.

## 1. Introduction

Peripheral inflammatory cytokines originating from infections in organs such as the lungs, liver, and intestines can disseminate via the bloodstream and vagal pathways [1]. The resultant systemic inflammatory response indirectly triggers brain nerve injury and leads to cognitive dysfunction, manifesting as memory impairment and confusion [2]. This represents a crucial mechanism through which local inflammation affects brain function. Angiotensin-converting enzyme 2 (ACE2), a component of the renin–angiotensin system (RAS), has emerged as a central player in inflammatory progression [3,4]. Through the ACE2-Ang-(1-7)-Mas axis, ACE2 exerts pleiotropic physiological activities, including vasodilation, anti-inflammatory effects, and tissue regeneration [5]. Currently available anti-inflammatory drugs can effectively upregulate ACE2 expression and activate the RAS axis, but their clinical use is often limited by adverse effects [6,7]. Consequently, naturally active molecules have garnered increasing attention [8]. For instance, marine foods are typically rich in proteins and unsaturated lipids, and epidemiological evidence suggests that regular consumption of healthy aquatic products is positively correlated with ameliorated inflammation and enhanced memory [9]. Moreover, our previous work employing surface plasmon resonance (SPR)-based screening successfully identified an oyster-derived peptide, KSVSPKFLTG, that binds to and activates ACE2 [10]. This peptide reduces the expression of pro-inflammatory factors in vivo and alleviates pulmonary inflammation. However, its potential role in modulating pneumonia-induced cognitive impairment remains unexplored. 

Notably, key components of the RAS, including ACE2, Ang II, and MAS1, are widely expressed in the brain, particularly in regions critically involved in cognition and motor function such as the hippocampus and substantia nigra [11,12]. ACE2 can directly degrade Ang II and generate Ang-(1-7), thereby rebalancing the RAS axis and mitigating neuronal injury [13]. Porel et al. elucidated that the Ang-(1-7)/Mas axis has neuroprotective effects and highlighted the potential of ACE inhibitor drugs to treat neurodegenerative diseases by regulating RAS balance [14]. Moreover, dysregulation of the brain RAS is highly correlated with dopaminergic neuron degeneration in Parkinson’s disease pathology, where the loss of these neurons reduces dopamine secretion and gives rise to classic symptoms, including bradykinesia and tremor [15,16]. These findings suggest that the oyster-derived anti-inflammatory peptide may exert neuroprotective effects by modulating the brain RAS balance. 

Given the growing consumer demand for health-promoting foods, there is increasing interest in the multi-dimensional and synergistic regulatory effects of food-derived components, along with deeper investigation into their mechanisms of action [17,18]. However, whether the oyster-derived anti-inflammatory peptide can also regulate RAS balance in neurons to confer neuroprotection remains to be elucidated. To address this, 1-methyl-4-phenylpyridinium (MPP^+^), a classic inducer that selectively enters dopaminergic neurons and inhibits mitochondrial complex I, was chosen to establish oxidative and inflammatory microenvironments [19]. This process is accompanied by intracellular iron accumulation, a marked increase in lipid peroxidation products, and exacerbation of neuronal ferroptosis [20,21]. In this study, we employed ferric ammonium citrate (FAC) and MPP^+^ to construct mild and acute models of dopaminergic neuronal injury. The effects of the oyster-derived ACE2-activating peptide were systematically evaluated by measuring reactive oxygen species (ROS) accumulation, loss of mitochondrial membrane potential, iron overload, and the expression of ferroptosis-related signaling factors. This study provides a solid theoretical foundation for the oyster-derived anti-inflammatory peptide as a novel RAS-modulating neuroprotective agent and simultaneously opens new avenues for the high-value utilization of marine biological resources such as oysters. 

## 2. Materials and Methods

### 2.1. Materials and Reagents

Lys-Ser-Val-Ser-Pro-Lys-Phe-Leu-Thr-Gly (KSVSPKFLTG, purity 98.0%, desalting) was obtained from Nanjing Peptide Valley Biotechnology Co., Ltd. (Nanjing, China) (Figure S1). ACE2 (SAB49072), Nrf2 (SAB14242), GPX4 (SAB49731), BCBP2 (SAB60205) and xCT (SAB56243) were purchased from Signalway Antibody (Greenbelt, MD, USA). SLC40A1 (A14884) and MAS1L (A16161) were purchased from ABclonal Technology (Wuhan, China). SHSY-5Y cells were obtained from Procell Biotechnology Co., Ltd. (CL-0208) (Wuhan, China). Reactive Oxygen Species (ROS) Detection Kit (S0033S) was purchased from Beyotime Biotechnology Co., Ltd. (Shanghai, China). The Prussian Blue Staining Kit (G1426) was obtained from Solarbio Science and Technology Co., Ltd. (Beijing, China). CheKine™ Micro Cell Total Iron Ion Content Assay Kit (KTB1114), Mitochondrial Membrane Potential Assay Kit (JC-1) (KTA4001), and CheKine™ Micro Lipid Peroxidation (MDA) Assay kit (KTB1050) were purchased from Abbkine Scientifc Co., Ltd. (Wuhan, China). Glutathione peroxidase 4 Assay Kit (H545-1-1) and Superoxide Dismutase (SOD) Assay Kit (A001-3-1) were purchased from Nanjing Jiancheng Bioengineering Institute (Nanjing, China). 1-Methyl-4-phenylpyridinium iodide (MPP^+^) (M875357) was obtained from Macklin Biochemical Technology Co., Ltd. (Shanghai, China). Ammonium ferric citrate (FAC)(A1495793) was purchased from Ambeed, Inc. (Buffalo Grove, IL USA). Cell Counting Kit-8 (CCK-8) (GK10001) was purchased from GlpBio (Montclair, CA, USA). 

### 2.2. Cell Cultures

SHSY-5Y cells were cultivated in MEM medium containing 10% fetal bovine serum and 1% penicillin−streptomycin solution in a humidified incubator (37 °C, 5% CO_2_ and 95% air). Then, 0.25% trypsin−EDTA was used to subculture cells when they reached 80 to 90% confluence. Cells within a passage number of 15 were used for experiments. All SHSY-5Y cells were divided into five groups: the healthy group (control), the model group (MPP^+^/FAC), the low-concentration peptide group (62.5 μM), the medium-concentration peptide group (125 μM), and the high-concentration peptide group (250 μM). Based on previous studies, the working concentration of MPP^+^ was set to 1 mM [22] and FAC to 100 μM [23], and these concentrations were consistently adopted throughout all subsequent experiments. For the control, healthy SHSY-5Y cells were cultured for 24 h. For the model group, MPP^+^/FAC were added based on the control and cultured for 24 h. To investigate the protective effects of the peptide, cells were first supplemented with peptide at final concentrations of 62.5 μM, 125 μM, and 250 μM and incubated at 37 °C. Afterwards, MPP^+^ and FAC were added to induce cell injury for 24 h. 

### 2.3. Determination of Cell Viability

To measure the cytotoxicity of MPP^+^, FAC, and KSVSPKFLTG peptide, SHSY-5Y cells were seeded in 96-well plates with a density of 1 × 10^4^ cells per well. After overnight incubation, the medium was replaced with fresh medium containing MPP^+^/FAC/KSVSPKFLTG peptide at different concentrations. KSVSPKFLTG was tested at a concentration gradient of 62.5, 125, 250, 500, and 1000 μM. After exposure for 24 h, the CCK-8 cell viability assay kit was employed according to the manufacturer’s instructions. The absorbance data were plotted using GraphPad Prism version 9.0 (GraphPad Software, San Diego, CA, USA). 

### 2.4. Intracellular ROS Fluorescence Imaging

SH-SY5Y cells were seeded in 24-well plates at 5 × 10^4^ cells/well and cultured for 24 h. After treatment with MPP^+^ and KSVSPKFLTG peptide for 24 h, cells were washed three times with PBS and incubated with 10 μM DCFH-DA for 30 min. Following another three PBS washes, ROS fluorescence images were captured using an inverted fluorescence microscope with fixed parameters (20× objective; excitation: 488 nm; emission: 530 nm). All images were quantitatively analyzed in a blinded manner with random sample renaming. ImageJ (win64) was used for background correction and calculation of single-cell mean fluorescence intensity. Multiple fields from three independent biological replicates were analyzed with sufficient cell numbers for statistical evaluation. 

### 2.5. Determination of Total Iron Ion Content

SH-SY5Y cells were seeded evenly in 12-well plates at 3 × 10^5^ cells per well and cultured for 24 h to adhere before drug treatment. After treatment, medium was removed, and cells were gently washed twice with pre-cooled PBS. Cells were scraped and centrifuged at 800× *g* for 5 min at 4 °C to collect pellets. Pellets were lysed, and supernatants were harvested after centrifugation. Absorbance was determined at 593 nm with a microplate reader. Total iron content was normalized to total protein quantified via the BCA assay, and relative cellular total iron levels were calculated. 

### 2.6. Prussian Blue Staining Assay

SH-SY5Y cells were seeded into 12-well plates at a density of 3 × 10^5^ cells per well and cultured for 24 h to achieve stable adhesion prior to drug intervention. After treatment, cells from five groups were rinsed twice with PBS and fixed with methanol for 5 min. Equal volumes of Perls stain A (3 mL) and Perls stain B (3 mL) supplied in the kit were mixed to prepare the Perls staining working solution. Cells were incubated with the working solution at 37 °C for 30 min, followed by washing with distilled water for 2 min. Subsequently, cells were counterstained with Perls counterstain for 1 min, rinsed with water, and observed under a microscope. 

### 2.7. Detection of Mitochondrial Membrane Potential (MMP)

MMP detection was conducted by the method of the mitochondrial membrane potential assay kit (JC-1). Cells were seeded in 24-well plates at 1 × 10^6^ cells/mL and treated following the same preliminary steps as method 2.2. The cells were washed once with PBS before being incubated with 10 μg/mL JC-1 staining solution for 20 min and washed twice with JC-1 staining buffer. Then, the stained cells were detected using a flow cytometer to record the JC-1 aggregates (585/590 nm) and JC-1 monomers (514/529 nm). 

### 2.8. Measurement of Glutathione Peroxidase 4 (GPX4)

Intracellular GPX4 activity was detected using a GPX4-specific kit (H545-1-1) from Nanjing Jiancheng Bioengineering Institute (Nanjing, China). SH-SY5Y cells were seeded at 3 × 10^5^ cells/well in 6-well plates and treated as described in Section 2.2. Cells were harvested, rinsed with PBS, lysed in cold extraction buffer on ice for 30 min, and centrifuged at 10,000× *g* for 10 min at 4 °C. The collected supernatants were quantified for total protein via BCA assay. After mixing with the reaction system and pre-incubation for 5 min at room temperature, the reaction was initiated with PCOOH substrate. The absorbance at 340 nm was recorded every 30 s for 5 min. GPX4 activity was calculated according to the kit instructions and normalized to total protein levels. 

### 2.9. Cellular Lipid Peroxidation (MDA) and Superoxide Dismutase (SOD) Assay

MDA and SOD, respectively, represent cellular lipid peroxidation and antioxidant levels. The BCA assay kit was utilized to quantify the protein concentrations. SHSY-5Y cells were seeded in 24-well plates at a density of 1 × 10^6^ cells/well. Different groups were subjected to peptide pre-treatment (62.5 μM, 125 μM, 250 μM) and MPP^+^ (1 mM) injury for 24 h. Trypsin was added to digest cells, and the cells were transferred to a 1.5 mL centrifuge tube. The MDA content and SOD activity were detected according to the manufacturers’ instructions. The supernatant (200 μL) from each tube was pipetted into a 96-well plate, and the absorbance of each well at 532 nm and 600 nm was measured with a microplate reader. 

### 2.10. Western Blot Analysis

SH-SY5Y cells were seeded into 12-well plates at 3 × 10^5^ cells per well and cultured for 24 h to stabilize adhesion before drug treatment. Post-treatment, target cells were harvested, and total protein was isolated using RIPA lysis buffer supplemented with protease inhibitors. Lysates were centrifuged at 14,000× *g* for 15 min at 4 °C, and protein concentrations were quantified via the BCA protein assay kit. Equal quantities of denatured proteins were separated by SDS-PAGE and transferred to PVDF membranes (Millipore, Billerica, MA, USA). Following blocking, membranes were incubated with primary antibodies (primary antibody cocktail for the target protein and GAPDH) at 4 °C overnight, then with HRP-conjugated secondary antibodies for 1 h at room temperature. Protein bands were visualized with an enhanced chemiluminescent substrate and scanned using OmegaLum G imaging software (version: 2.0.1027.0; Aplegen, San Francisco, CA, USA). Bands corresponding to the target protein and GAPDH were visualized on the same PVDF membrane after chemiluminescence detection. 

### 2.11. Statistical Analysis

Each experiment was repeated three times. The symbol *n* denotes the number of independent biological replicates. Technical replicates within a single biological replicate were not treated as independent samples and excluded from statistical analysis. Data are expressed as mean ± standard deviation (SD). Statistical analyses were performed using GraphPad Prism 9.0. Intergroup differences were analyzed by one-way ANOVA followed by LSD post hoc test. *p* < 0.05 was considered statistically significant. The proportions of cell populations obtained from JC-1 flow cytometry assays were analyzed following the identical statistical framework described above. 

## 3. Results

### 3.1. KSVSPKFLTG Peptide Alleviates Oxidative Damage of SHSY-5Y Cells

**Figure 1:**
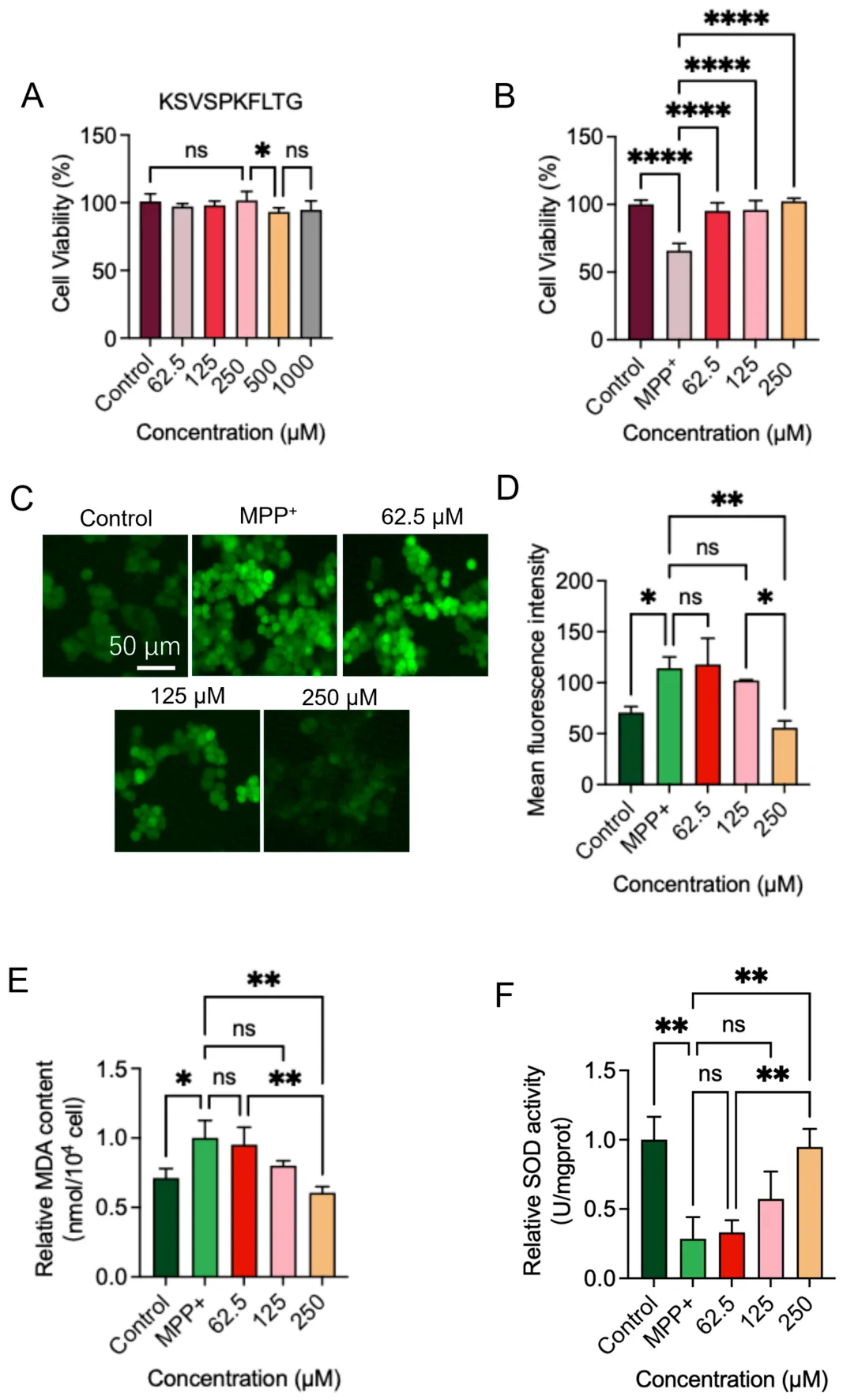
Effects of KSVSPKFLTG peptide on the antioxidant defense of SHSY-5Y cells. (**A**) Effects of different concentrations of KSVSPKFLTG on cell viability. (**B**) Alleviative effects of KSVSPKFLTG at different concentrations on cellular injury. (**C**) Fluorescence images of ROS in SH-SY5Y cells under different treatments (scale bar = 50 μm). (**D**) Quantitative analysis of ROS fluorescence intensity in SH-SY5Y cells under different treatments. (**E**) The relative MDA content in SHSY-5Y cells. (**F**) The relative SOD activity in SHSY-5Y cells. All experiments were performed with *n* = 6 biological replicates. The significance of the ANOVA analysis is expressed as * *p* < 0.05, ** *p* < 0.01 and **** *p* < 0.0001 (ns, not significant).

To explore the optimal concentration of KSVSPKFLTG, this study conducted experimental determination with increasing peptide concentrations. As shown in Figure 1A, the viability of SHSY-5Y cells treated with KSVSPKFLTG (62.5, 125, 250, and 200 μM) has no significant difference from that of the control group. The viability of SHSY-5Y cells in the 250 μM peptide group was significantly different from that of the 500 μM group (*p* < 0.05). So, the three concentrations of 62.5, 125, and 250 μM were selected for subsequent experiments. 

Under MPP^+^ impairment, the cell viability decreased by 34.15% compared to that of the control group (*p* < 0.0001). The cell viability significantly increased with peptide treatment (*p* < 0.0001), reaching the peak at the 250 μM group (Figure 1B). The results in Figure 1C,D showed that the protective effect of the peptide was related to its regulation of intracellular ROS levels. Visualization by the DCFH-DA fluorescent probe, cells in the MPP^+^ group showed high fluorescence intensity, indicating a large accumulation of intracellular ROS. After treatment with the peptide, the fluorescence intensity in SHSY-5Y cells showed a downward trend with the increase in KSVSPKFLTG concentration. Quantitative statistics showed the fluorescence intensity of SHSY-5Y cells in the 250 μM group was significantly lower than that in the MPP^+^ group (*p* < 0.01). The above results confirmed that the peptide reduced excessive accumulated ROS in SHSY-5Y cells. 

The intracellular lipid peroxidation level and antioxidant enzyme content were determined (Figure 1E). Compared with the control group, the MDA content of the MPP^+^ group was significantly increased (*p* < 0.05), indicating that MPP^+^ induced intracellular lipid peroxidation. The MDA content showed a significant downward trend with 250 μM peptide treatment (*p* < 0.01). Moreover, compared with the control group, MPP^+^ treatment significantly reduced the SOD activity from 1.00 to 0.29 (*p* < 0.01), resulting in a dramatic decrease in antioxidant defense capacity (Figure 1F). The 250 μM group had significantly increased SOD activity compared to the MPP^+^ group (*p* < 0.01). In summary, KSVSPKFLTG scavenged ROS and inhibited lipid peroxidation, thereby alleviating MPP^+^-mediated cellular oxidative damage. 

### 3.2. KSVSPKFLTG Peptide Improves MMP via an Antioxidation-Dependent Pathway

**Figure 2:**
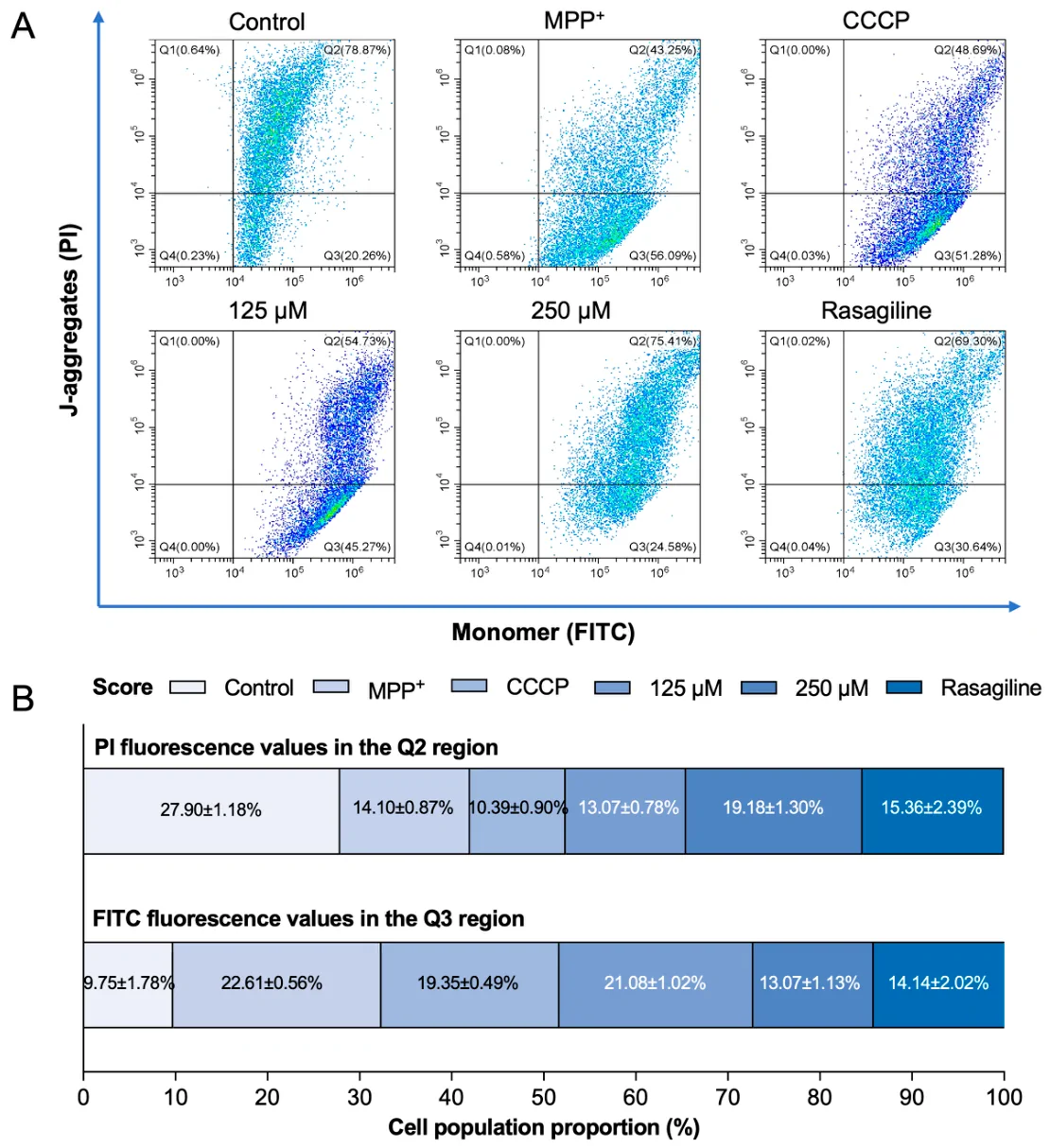
Detection of mitochondrial membrane potential upon KSVSPKFLTG peptide treatment using JC-1 staining. (**A**) Representative JC-1 flow cytometry scatter plots. (**B**) Quantification of the percentage of cells with high JC-1 aggregate (red, Q2) fluorescence and high JC-1 monomer (green, Q3) fluorescence across six experimental groups. Data are presented as mean ± SD; *n* = 3 independent biological replicates.

JC-1 fluorescent probe combined with flow cytometry was employed to investigate MMP in MPP^+^-induced SH-SY5Y cells. As shown in Figure 2A, cells with normal MMP were distributed in the Q2 region with high J-aggregate fluorescence, whereas cells with dissipated MMP accumulated in the Q3 region with abundant monomeric JC-1 fluorescence. The uncoupler CCCP served as a negative control to induce MMP loss, and rasagiline was used as a positive control for MMP restoration. Healthy cells in the control group were mainly distributed in Q2 (27.90 ± 1.18%), with only 9.75 ± 1.78% of cells located in Q3 (Figure 2B), indicating intact MMP. In contrast, more cells in the MPP^+^ group shifted to Q3, and the percentage of Q2 cells decreased to 14.10 ± 0.87%. These data demonstrate that MPP^+^ markedly triggers MMP decline and mitochondrial injury. After treatment with KSVSPKFLTG, the proportion of Q2 cells displayed a concentration-dependent recovery trend. The percentage of Q2 cells in the 250 μM group increased to 19.18 ± 1.30% compared with the MPP^+^ group (14.10 ± 0.87%) and CCCP group (10.39 ± 0.90%), approaching the level of the rasagiline group (15.36 ± 2.39%). Collectively, these results indicate that KSVSPKFLTG effectively reverses MPP^+^-provoked MMP reduction and alleviates mitochondrial damage. 

### 3.3. KSVSPKFLTG Peptide Modulates Iron Deposition in SHSY-5Y Cells

**Figure 3:**
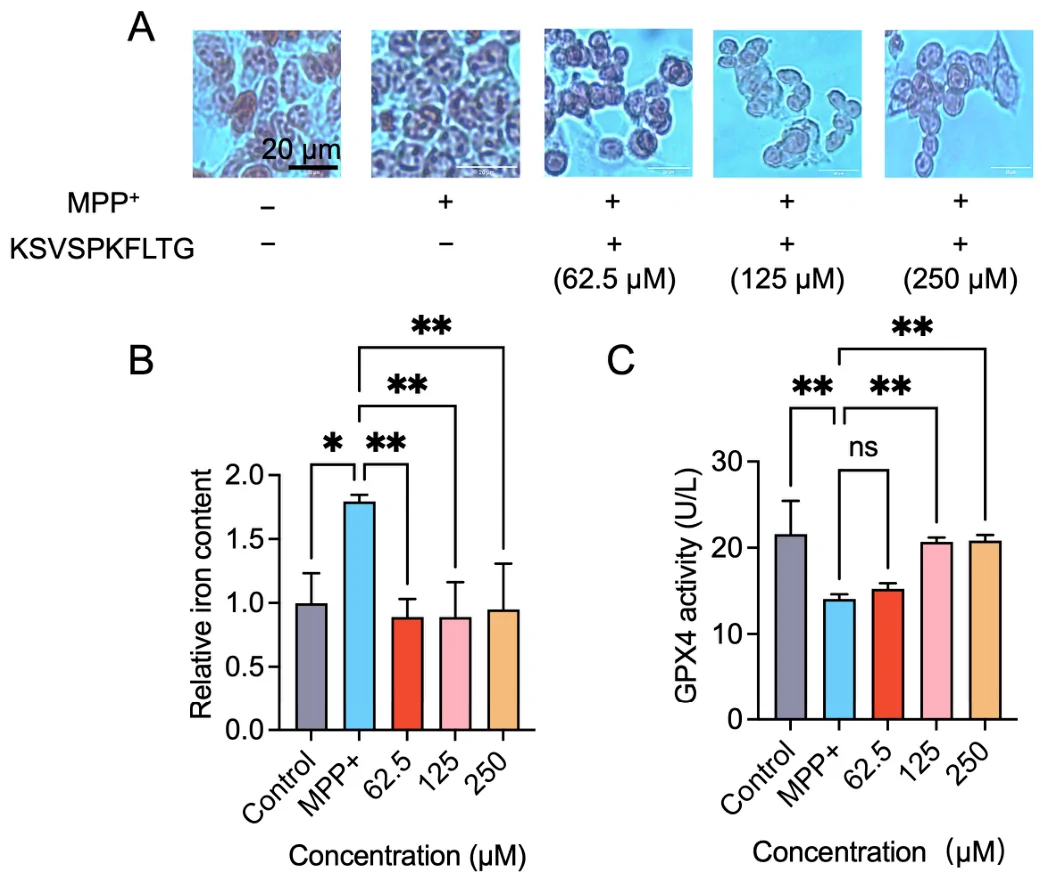
Cytosolic iron deposition status of SHSY-5Y cells. (**A**) Prussian blue staining of SHSY-5Y cells (scale bar = 20 μm). (**B**) Determination of total cytosolic iron content in SHSY-5Y cells. (**C**) Cytosolic GPX4 activity assay in SHSY-5Y cells. All experiments were performed with *n* = 3 biological replicates. The significance of the ANOVA analysis is expressed as * *p* < 0.05, ** *p* < 0.01 (ns, not significant).

Prussian blue staining was employed to observe the level of intracellular iron deposition, which is a core factor for ferroptosis. As shown in Figure 3A, an iron deposition plaque was observed in the MPP^+^ group, whereas the iron deposition levels of SHSY-5Y cells in the 125 μM and 250 μM groups treated with the peptide showed an obvious decrease. Moreover, the quantitative determination of total intracellular iron content showed that the damage of MPP^+^ significantly aggravated the abnormal accumulation of intracellular free iron (*p* < 0.05) (Figure 3B). The peptide significantly reduced abnormal iron accumulation by regulating the oxidative microenvironment (*p* < 0.01). In addition, compared with the MPP^+^ group, GPX4 activity in MPP^+^-induced SHSY-5Y cells was significantly enhanced with 125 μM and 250 μM peptide treatment (*p* < 0.01). The above results indicated that KSVSPKFLTG significantly reduces the iron deposition in SHSY-5Y cells, thereby alleviating ROS accumulation and oxidative stress damage caused by the Fenton reaction. 

### 3.4. KSVSPKFLTG Inhibits FAC-Induced Imbalance of Iron Homeostasis and Antioxidant Defense in SHSY-5Y Cells via Activating RAS Axis

**Figure 4:**
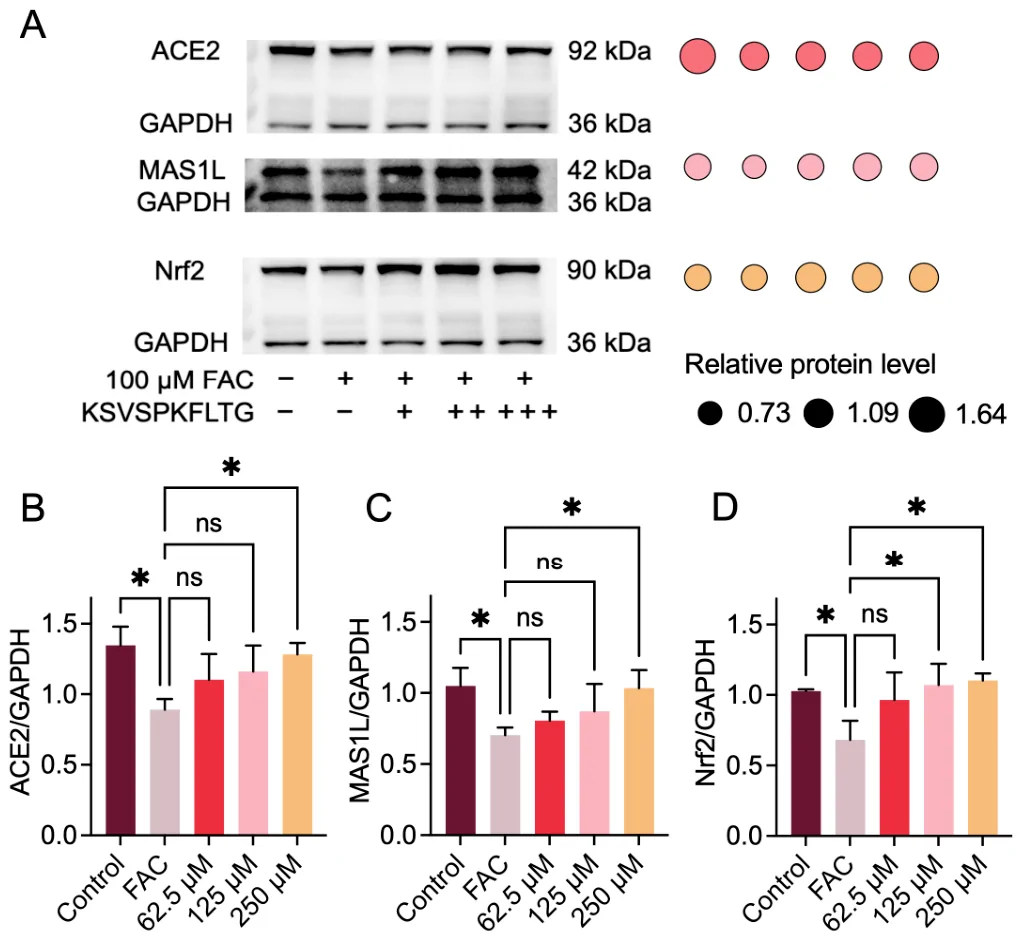
Western blot analysis of the protein expression of ACE2, MAS1L, and Nrf2. (**A**) Western blot bands for different proteins. The size of bubbles with the same color corresponds to the relative grayscale values of the target proteins in their respective groups. (**B**–**D**) The expression levels of ACE2, MAS1L, and Nrf2 in SHSY-5Y cells. All experiments were performed with *n* = 3 biological replicates. The significance of the ANOVA analysis is expressed as * *p* < 0.05 (ns, not significant).

To examine whether this ACE2-activated peptide improves intracellular iron homeostasis and oxidative stress in SHSY-5Y cells, FAC was used to create an iron-overload state in SHSY-5Y cells. As shown in the Western blot results of Figure 4A,B, the ACE2 content in the FAC group was significantly decreased by 33.83 ± 7.57% compared with the control group (*p* < 0.05). The ACE2 content in SHSY-5Y cells was relatively recovered after peptide treatment, which was significantly higher (1.28 ± 0.08%) in the 250 μM group than that of the FAC group (0.89 ± 0.08%) (*p* < 0.05). In addition, the MAS1L content of SHSY-5Y cells in the 250 μM group (1.03 ± 0.13%) was also significantly upregulated compared to that in the FAC group (0.70 ± 0.05%) (*p* < 0.05). Moreover, Nrf2 directs intracellular redox homeostasis and participates in detoxification and anti-inflammatory processes [24]. Compared with the FAC group, transcription factor Nrf2 expression was significantly upregulated in the 125 μM and 250 μM peptide groups (*p* < 0.05), reaching levels comparable to the control group. Collectively, KSVSKFLTG elevates ACE2 and MAS1L, core components of the protective RAS axis. Consistent with known studies [10], this peptide directly binds ACE2 to boost its intrinsic catalytic activity and simultaneously increases cellular ACE2 protein abundance. The combination of higher ACE2 abundance and enhanced enzyme activity promotes Ang II degradation to Ang-(1–7), triggering downstream Nrf2-mediated antioxidant responses, rebalancing the RAS axis, and alleviating neuronal injury. These results indicate that KSVSKFLTG modulates the protective RAS axis to strengthen cellular antioxidant defense. 

**Figure 5:**
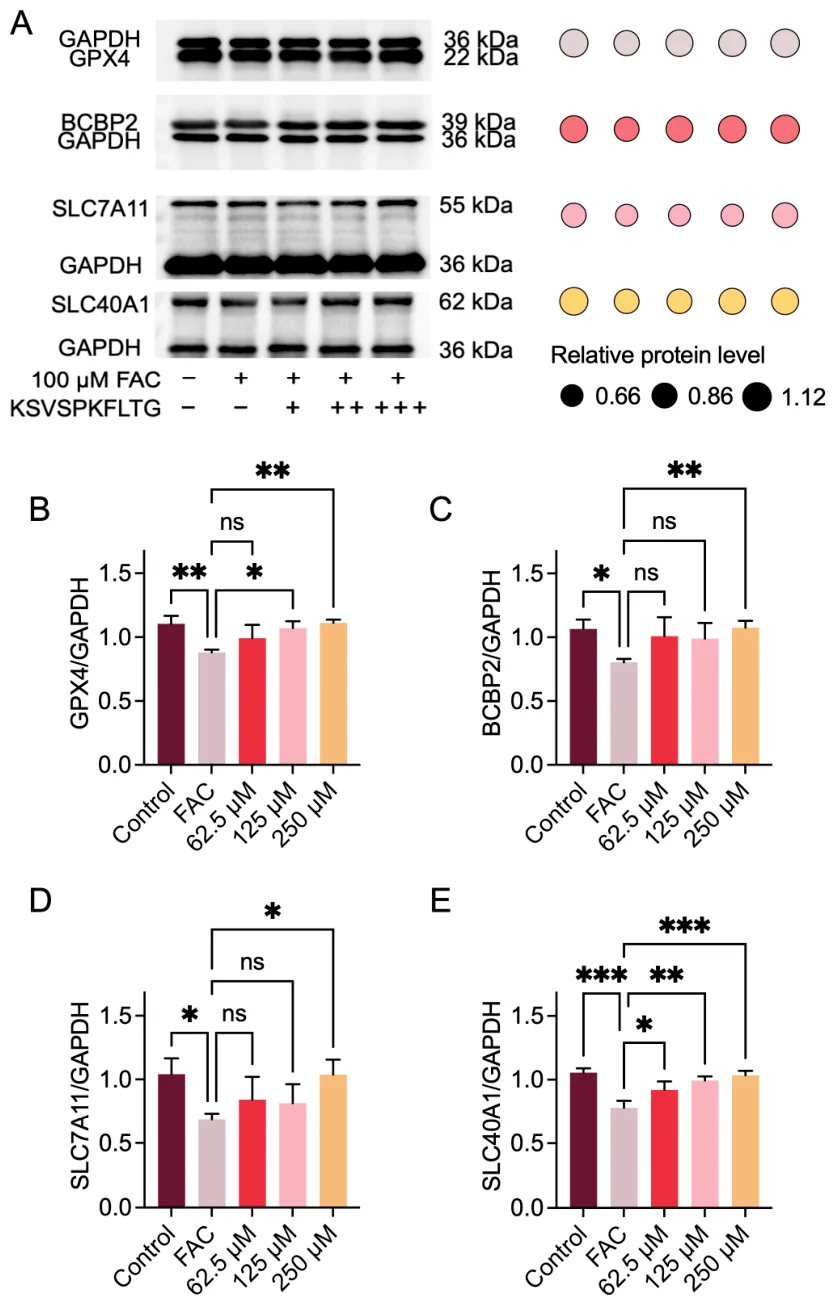
Western blot analysis of the protein expression of GPX4, BCBP2, SLC7A11, and SLC40A1. (**A**) Western blot bands for different proteins. The size of bubbles with the same color corresponds to the relative grayscale values of the target proteins in their respective groups. (**B**–**E**) The expression levels of GPX4, BCBP2, SLC7A11, and SLC40A1 in SHSY-5Y cells. All experiments were performed with *n* = 3 biological replicates. The significance of the ANOVA analysis is expressed as * *p* < 0.05, ** *p* < 0.01 and *** *p* < 0.001 (ns, not significant).

The expression levels of ferroptosis-related protein markers, including GPX4, BCBP2, SLC7A11, and SLC40A1, were quantified by Western blot. As shown in Figure 5, the contents of GPX4 and SLC40A1 in SHSY-5Y cells in the 250 μM groups were significantly higher than those in the FAC group (GPX4: *p* < 0.01, SLC40A1: *p* < 0.001) after peptide treatment. Moreover, the contents of BCBP2 and SLC7A11 in SHSY-5Y cells treated with 250 μM peptide (BCBP2: 1.08 ± 0.05, SLC7A11: 1.03 ± 0.13) were significantly higher than those in the FAC group (BCBP2: 0.80 ± 0.03, *p* < 0.01, SLC7A11: 0.68 ± 0.05, *p* < 0.05) (Figure 5B–E). These results indicate that the ACE2-activated peptide improves intracellular iron homeostasis and oxidative stress in SHSY-5Y cells. 

### 3.5. ACE2-Activated Peptide Inhibits MPP^+^-Induced Nerve Impairment

**Figure 6:**
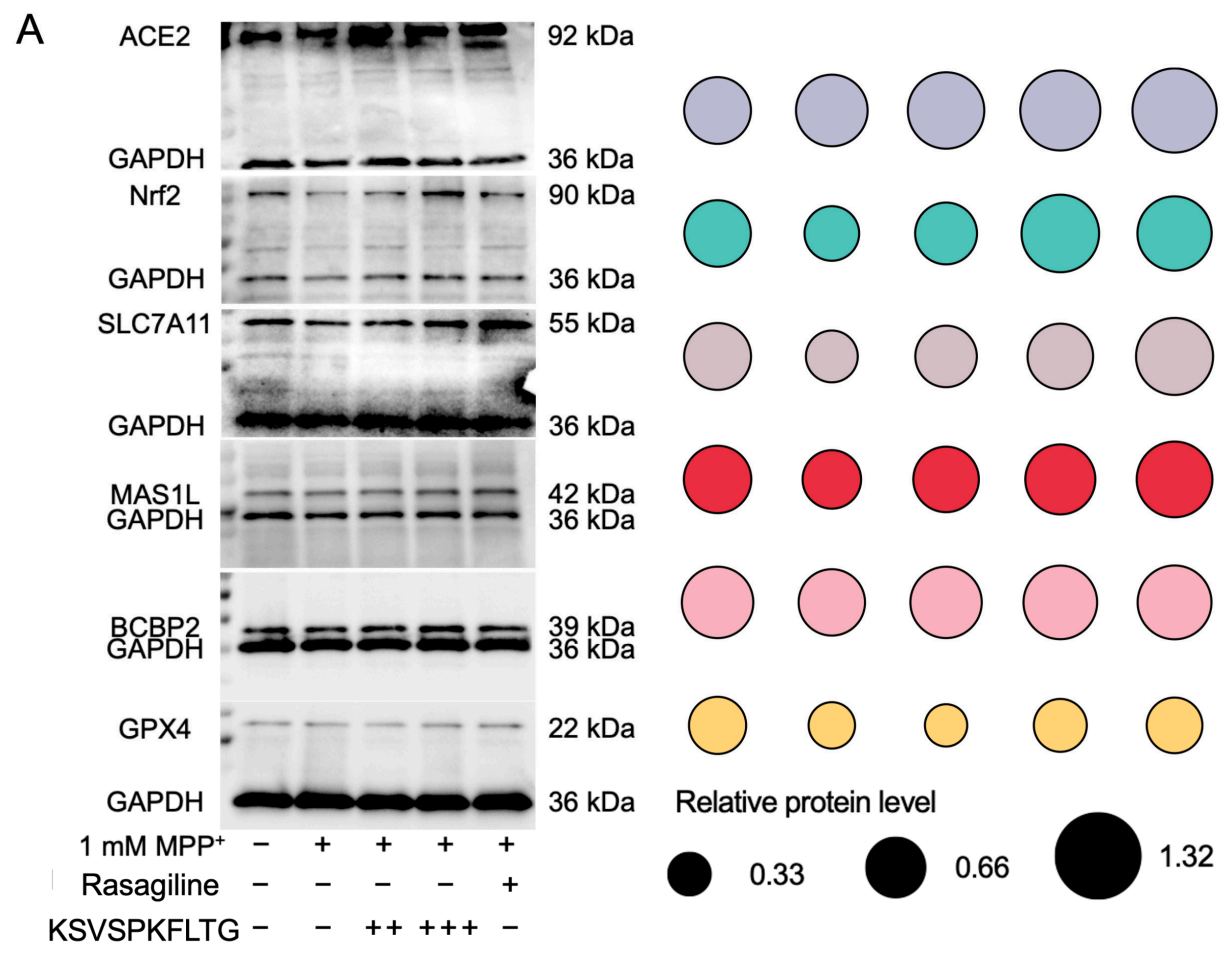

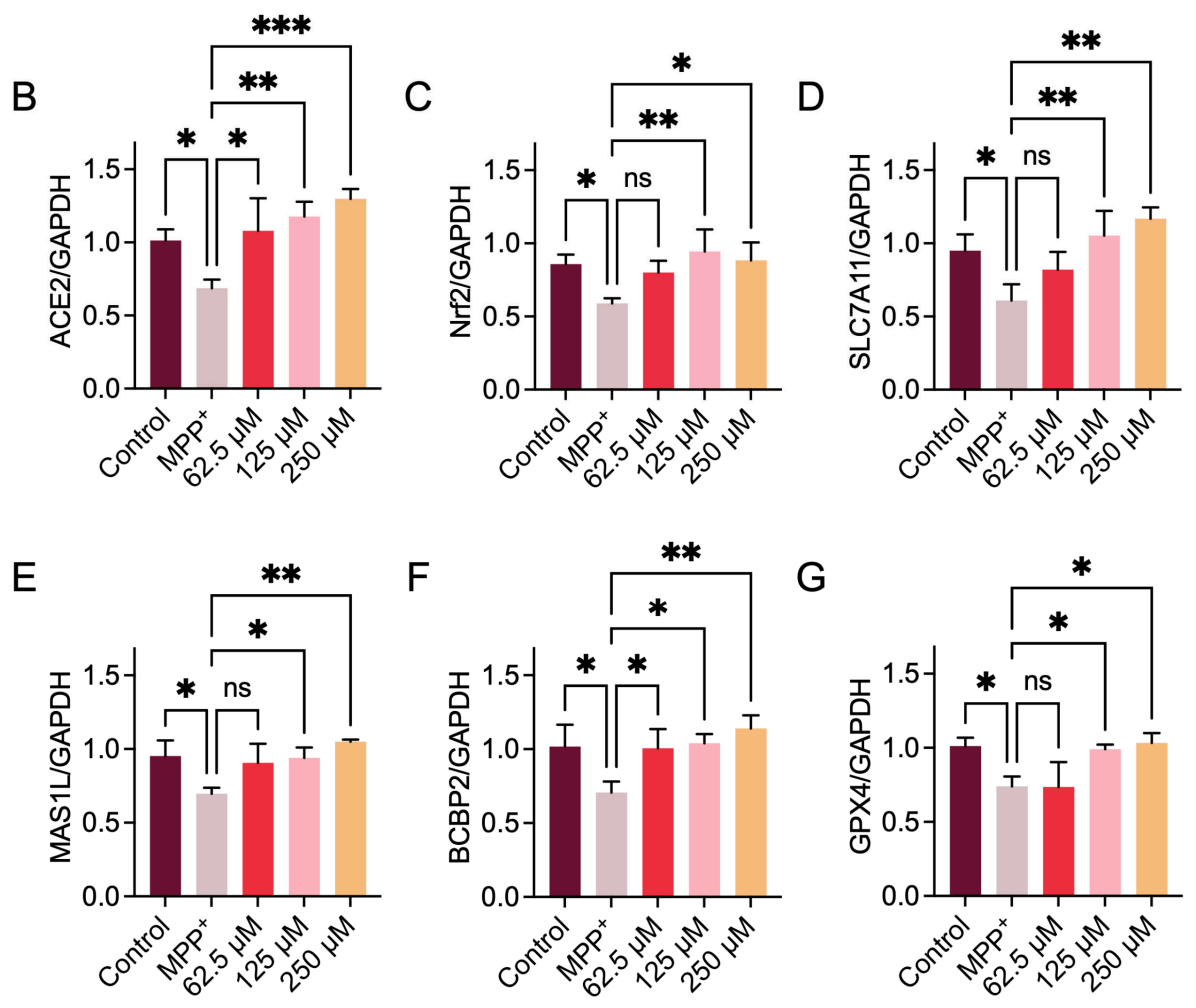
Western blot analysis of the protein expression of ACE2, Nrf2, SLC7A11, MAS, BCBP2, and GPX4. (**A**) Different Western blot bands. The size of bubbles with the same color corresponds to the relative grayscale values of the target proteins in their respective groups. (**B**–**G**) The expression levels of ACE2, Nrf2, SLC7A11, MAS, BCBP2 and GPX4 in SHSY-5Y cells. All experiments were performed with *n* = 3 biological replicates. The significance of the ANOVA analysis is expressed as * *p* < 0.05, ** *p* < 0.01 and *** *p* < 0.001 (ns, not significant).

To further confirm that the ACE2-activated peptide maintained the antioxidant microenvironment of neurons, the expression levels of ACE2, Nrf2, SLC7A11, MAS1L, BCBP2, and GPX4 in MPP^+^-induced SHSY-5Y cells were re-confirmed by Western blot. Also, Rasagiline was utilized as a positive control. Compared with the control group, the expression level of ACE2 in the MPP^+^ group was downregulated by 26.18 ± 7.30% relative to that of the control group (Figure 6A,B). The treatment of KSVSPKFLTG peptide significantly upregulated ACE2 levels in the 250 μM (*p* < 0.001) and rasagiline groups (*p* < 0.01), indicating the ability of this peptide to activate the RAS axis. In addition, Nrf2 and GPX4 in the MPP^+^ group were downregulated by 20.96 ± 1.28% and 24.05 ± 4.17%, respectively (Figure 6C,G). After 250 μM peptide treatment, the levels of Nrf2 and GPX4 in SHSY-5Y cells (Nrf2: 0.88 ± 0.12, GPX4: 1.03 ± 0.07) were significantly higher than those in MPP^+^ group (Nrf2: 0.59 ± 0.04, *p* < 0.05, GPX4: 0.75 ± 0.07, *p* < 0.05). This suggested the ACE2-activated peptide may promote the expression of antioxidant factors and maintain the activity of antioxidant enzymes via activating the RAS axis. Moreover, the levels of SLC7A11, MAS1L and BCBP2 in the MPP^+^ group were downregulated by 21.27 ± 3.52%, 21.10 ± 1.35% and 26.86 ± 8.35% relative to those in the control group (Figure 6D–F), which were significantly increased in the 250 μM peptide (*p* < 0.05) and rasagiline groups (*p* < 0.01) compared with those in the MPP^+^ group. Western blot results showed that treatment with the peptide KSVSPKFLTG upregulated the protein expression of ACE2, MAS1L, Nrf2, SLC7A11, GPX4, and BCBP2. ACE2 and MAS1L are key molecules of the protective RAS axis, and their increased expression implies enhanced activity of the intracellular protective ACE2/Ang-(1–7)/MAS1L signaling cascade [10]. Enhanced protective RAS signaling further cooperates with the Nrf2 antioxidant pathway to elevate SLC7A11 and GPX4 levels. In addition, the upregulation of BCBP2 sustains cellular homeostasis, and these combined effects mitigate MPP^+^-induced neuronal injury. 

## 4. Discussion

The present study demonstrates that KSVSPKFLTG, a peptide derived from *Magallana gigas* previously identified as an ACE2-binding ligand [10], exerts significant neuroprotective effects against MPP^+^/FAC-induced cytotoxicity in SHSY-5Y cells. Mechanistically, this peptide not only attenuates oxidative stress and mitochondrial dysfunction but also mitigates ferroptosis via activating the RAS, particularly the ACE2-Ang-(1-7)-Mas axis. These findings extend our prior observations of its anti-inflammatory properties in pulmonary tissues and position this peptide as a multitargeted bioactive candidate for neurological intervention. Given the rising interest in food-derived bioactives for managing neurodegenerative conditions, our results offer a compelling rationale for further exploring oyster peptides as modulators of brain RAS homeostasis. 

The RAS is increasingly recognized as a key regulator of neuroinflammation and dopaminergic survival. While the classical ACE-Ang II-AT1R axis promotes oxidative injury [25,26], the counter-regulatory ACE2-Ang-(1-7)-Mas arm confers neuroprotection through vasodilation, anti-apoptotic signaling, and mitochondrial preservation [17,27]. In our model, MPP^+^ and FAC challenge markedly downregulated ACE2 and MAS1L expression, indicating a shift toward a pro-oxidant RAS phenotype. KSVSPKFLTG treatment reversed these changes, restoring ACE2 and MAS1L levels in a dose-responsive manner. This reactivation of the protective RAS axis likely underlies the peptide’s capacity to suppress ROS burst and preserve mitochondrial membrane potential, aligning with emerging evidence that RAS rebalancing mitigates dopaminergic degeneration in Parkinson’s disease models [15]. 

Oxidative stress is a hallmark of MPP^+^ neurotoxicity [28], driven by mitochondrial complex I inhibition and subsequent electron leakage. Our data corroborate that MPP^+^ exposure leads to pronounced ROS accumulation and lipid peroxidation, reflected by elevated MDA and depleted SOD activities. KSVSPKFLTG effectively curtailed these alterations, reducing ROS burden and restoring SOD to near-control levels. Notably, the peptide’s antioxidant action was concentration-dependent, with the 250 μM group showing the most robust protection. This effect was not merely scavenging-based, as the peptide concurrently upregulated Nrf2, a master transcriptional regulator of antioxidant enzymes. Nrf2 activation is a well-established consequence of RAS activation, suggesting that KSVSPKFLTG orchestrates a coordinated antioxidant response upstream of direct radical neutralization. 

Mitochondrial dysfunction constitutes another critical node in MPP^+^ pathogenesis. The collapse of MMP is an early event that commits cells to apoptotic or ferroptotic death. Using JC-1 flow cytometry, we observed that MPP^+^ shifted the majority of cells toward low MMP, while KSVSPKFLTG preserved the high-MMP population in a manner comparable to rasagiline, a standard neuroprotective agent. This mitochondrial protection likely stems from reduced ROS-mediated opening of the mitochondrial permeability transition pore and improved electron transport chain efficiency. Since ACE2 is expressed on mitochondrial membranes in dopaminergic neurons, the peptide may directly influence mitochondrial RAS signaling, an intriguing possibility that warrants further investigation using subcellular fractionation and respirometry. 

Ferroptosis, a non-apoptotic cell death modality driven by iron-dependent lipid peroxidation, has gained traction as a pathogenic mechanism in neurodegenerative diseases [29]. Our study provides multiple lines of evidence that KSVSPKFLTG counteracts ferroptotic cascades. First, the peptide reduced intracellular iron deposition, as visualized by Prussian blue staining and quantified by total iron content assays. Second, it upregulated GPX4, the central phospholipid hydroperoxidase that curtails ferroptosis [30]. Third, it enhanced SLC7A11 (xCT), the cystine/glutamate antiporter that supplies cysteine for glutathione synthesis, thereby sustaining GPX4 activity. Fourth, it increased BCBP2, a ferritinophagy regulator that limits labile iron pools, and SLC40A1, the sole cellular iron exporter, facilitating iron efflux. This multi-pronged modulation of iron homeostasis underscores a holistic cytoprotective strategy that distinguishes KSVSPKFLTG from conventional iron chelators. 

The interplay between RAS activation and ferroptosis suppression is a novel finding of this work. While ACE2 has been predominantly studied in the context of inflammation and vasodilation, its role in iron metabolism is underexplored. Our results suggest that ACE2 activation, possibly through Ang-(1-7)-Mas signaling, converges on the Nrf2 pathway, which transcriptionally regulates both antioxidant enzymes and iron-sequestering proteins. Nrf2 target genes include FTH1 and SLC40A1, all of which participate in iron detoxification. Thus, KSVSPKFLTG may function as a RAS-dependent Nrf2 agonist, integrating redox and iron regulatory networks. This concept is supported by the observed upregulation of Nrf2 and its downstream effectors following peptide treatment, reinforcing the therapeutic potential of targeting the RAS-Nrf2-ferroptosis axis. 

Comparing the MPP^+^ and FAC models, we noted subtle differences in the magnitude of peptide response. While both models recapitulated oxidative and ferroptotic features, FAC-induced iron overload may more directly challenge iron efflux mechanisms, whereas MPP^+^ primarily disrupts mitochondrial respiration, leading to secondary iron dysregulation via ROS-mediated ferritin damage. KSVSPKFLTG proved effective in both contexts, indicating that its actions are not confined to a single upstream insult. This broad-spectrum efficacy is clinically appealing, as neurodegenerative processes rarely involve a single pathogenic trigger. The peptide’s ability to activate RAS while simultaneously boosting GPX4 and SLC40A1 implies that it addresses both the cause and consequence of ferroptotic stress, a feature that synthetic drugs often lack due to their narrow target specificity. 

Despite these encouraging results, several limitations must be acknowledged. First, all experiments were performed using undifferentiated SH-SY5Y cells. This cell line exhibits stable proliferation, straightforward culture conditions, and endogenous ACE2 expression, making it suitable for preliminary mechanistic exploration in the present study. Nevertheless, undifferentiated SH-SY5Y cells originate from neuroblastoma and remain immature. This homogeneous single-cell model cannot fully recapitulate the complex intercellular interactions within the brain microenvironment [19]. Second, the precise molecular interaction between KSVSPKFLTG and ACE2—whether allosteric modulation or direct catalytic enhancement—requires structural elucidation via crystallography or molecular dynamics simulations. Third, the in vivo pharmacokinetic profiles, tissue distribution, and blood–brain barrier penetration capacity of KSVSPKFLTG have not yet been characterized. Low systemic bioavailability and limited blood–brain barrier permeability following peripheral administration may restrict its therapeutic efficacy, which requires further validation in animal models. Future strategies, including peptide cyclization and conjugation with brain-targeted moieties via chemical modification, can be adopted to improve brain accumulation. Intranasal delivery and brain-targeted nanocarrier systems can also bypass the barrier and enhance peptide stability. These approaches will facilitate further evaluation of the in vivo therapeutic potential of KSVSPKFLTG. Fourth, long-term toxicity and off-target effects need careful evaluation before considering clinical applications. Addressing these gaps will be critical for advancing KSVSPKFLTG toward preclinical development as a neuroprotective nutraceutical or pharmaceutical lead. 

## 5. Conclusions

In this study, the ACE2-activated peptide KSVSPKFLTG derived from *Magallana gigas* demonstrated significant neuroprotective effects against MPP^+^/FAC-induced injury in SHSY-5Y cells. This peptide effectively reduced intracellular ROS accumulation, enhanced antioxidant defenses, ameliorated the loss of MMP, and mitigated aberrant iron deposition. Moreover, KSVSPKFLTG upregulated the expression of ACE2 and its downstream effector MAS1L, thereby activating the renin–angiotensin system (RAS). This activation further promoted Nrf2-mediated antioxidant responses and enhanced the expression of ferroptosis-related protective proteins, including GPX4, SLC7A11, BCBP2, and the iron exporter SLC40A1. Collectively, these findings reveal that peptide KSVSPKFLTG protects neuronal cells from oxidative stress and ferroptosis by activating the RAS axis. It reveals a concurrent neuroprotective effect of this peptide in response to in vivo inflammation. 

## Data Availability

The data are not publicly available due to the excessive size of raw experimental datasets preventing packaging and upload to public repositories and portions of the data relating to our group’s ongoing, unpublished research. The datasets and materials used and/or analyzed during the current study are available from the corresponding author upon reasonable request.

## Supplementary Materials

The following supporting information can be downloaded at https://www.mdpi.com/article/10.3390/antiox15080992/s1.

## Abbreviations

The following abbreviations are used in this manuscript:

ACE2	Angiotensin-converting enzyme 2
RAS	Renin–angiotensin system
SPR	Surface plasmon resonance
MPP^+^	1-methyl-4-phenylpyridinium
FAC	Ferric ammonium citrate
ROS	Reactive oxygen species
MDA	Lipid peroxidation
SOD	Superoxide dismutase
MMP	Mitochondrial membrane potential
GSH-Px	Glutathione peroxidase
