# Supplementary material for: Neuroprotective Potential of Peptide KSVSPKFLTG from Magallana gigas via Activating the ACE2/Ang-(1-7)/Mas Axis in SHSY-5Y Cells
Source: Antioxidants (Basel). 2026 Aug 10;15(8):992. doi: 10.3390/antiox15080992 (PMC13509928; doi:10.3390/antiox15080992)
Supplement: Supplementary file 1 [file antioxidants-15-00992-s001.zip › antioxidants-4442814-supplementary.pdf]

Supporting Information

Neuroprotective Potential of Peptide KSVSPKFLTG from *Magallana gigas* via Activating the ACE2/Ang-(1-7)/Mas Axis in SHSY-5Y Cells

Junlong Lai[1,†], *Zhiwei Zheng*[1,†], *Jialong Gao*[1,2,3], *Zhihong Zheng*[1,2,3], *Haisheng Lin*[1,2,3], *Zhongqin Chen*[1,2,3], *Guoping Zhu*[1,2,3], *Wenhong Cao*[1,2,3], *Xiaoyu Xia*[1,2,3*]

[1] College of Food Science and Technology, Guangdong Ocean University, Zhanjiang 524088, China; zzh617@gdou.edu.cn (Z.Z.)

[2] Shenzhen Institute of Guangdong Ocean University, Shenzhen 518108, China

[3] National Research and Development Branch Center for Shellfish Processing (Zhanjiang), Guangdong Provincial Key Laboratory of Aquatic Products Processing and Safety, Guangdong Provincial Engineering Technology Research Center of Seafood, Zhanjiang 524088, China

*Corresponding Author: Xiaoyu Xia

*Email: xiaxiaoyu@gdou.edu.cn

1. Materials and Methods

Mass spectrometric identification of KSVSPKFLTG peptide. The peptide was obtained through solid-phase synthesis with assistance from Nanjing Peptide Valley Biotechnology Co., Ltd. Then it was identified through LC-MS/MS (Thermo Fisher Scientific, USA). The mobile phase was composed of buffer A (0.1% formic acid in water) and buffer B (0.1% formic acid in acetonitrile). Chromatographic separation was carried out at a fixed flow rate of 0.2 mL/min for 1 min. Automatic gain control (AGC) was enabled using the Orbitrap, and ions with controlled accumulation levels between $1e^5$ and $3e^6$ were selected for subsequent secondary scanning to facilitate identification. The ACE2-activating peptide was identified with an m/z of 532.76, and its amino acid sequence was automatically matched against an oyster protein database.

Purity determination of the KSVSPKFLTG peptide. The purity of the peptide was determined by high-performance liquid chromatography (HPLC) using mobile phase A (acetonitrile containing 0.1% trifluoroacetic acid) and mobile phase B (aqueous solution containing 0.1% trifluoroacetic acid). The gradient elution program was set as follows: 0.01 min, 5% A and 95% B; 25 min, 50% A and 50% B; 30 min, 90% A and 10% B. The flow rate was maintained at 1.0 mL/min, the injection volume was 20 μL, and the detection wavelength was 214 nm. The HPLC-measured purity of the peptide exceeded 98%.

2. Supporting figures

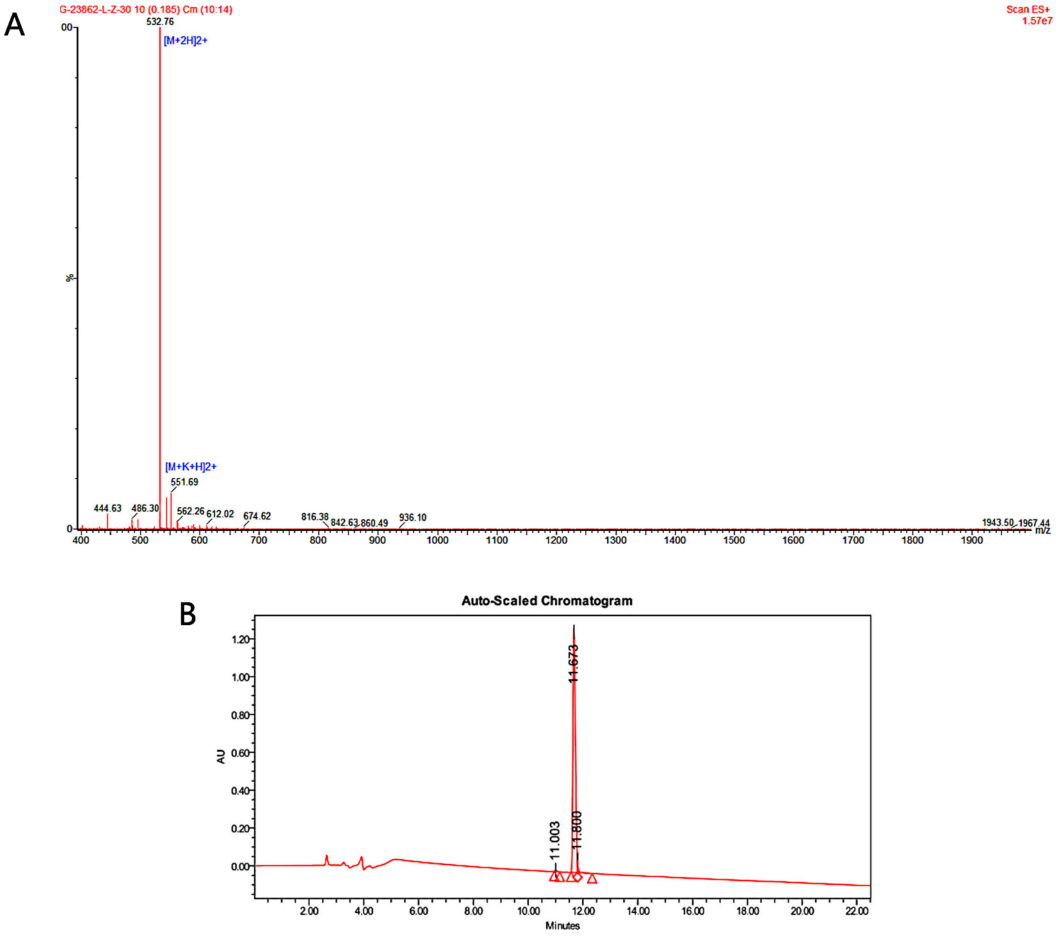

Figure S1. Sequence identification and purity analysis of the ACE2 activating peptide KSVSPKFLTG.

3. Raw images of Western blotting data.

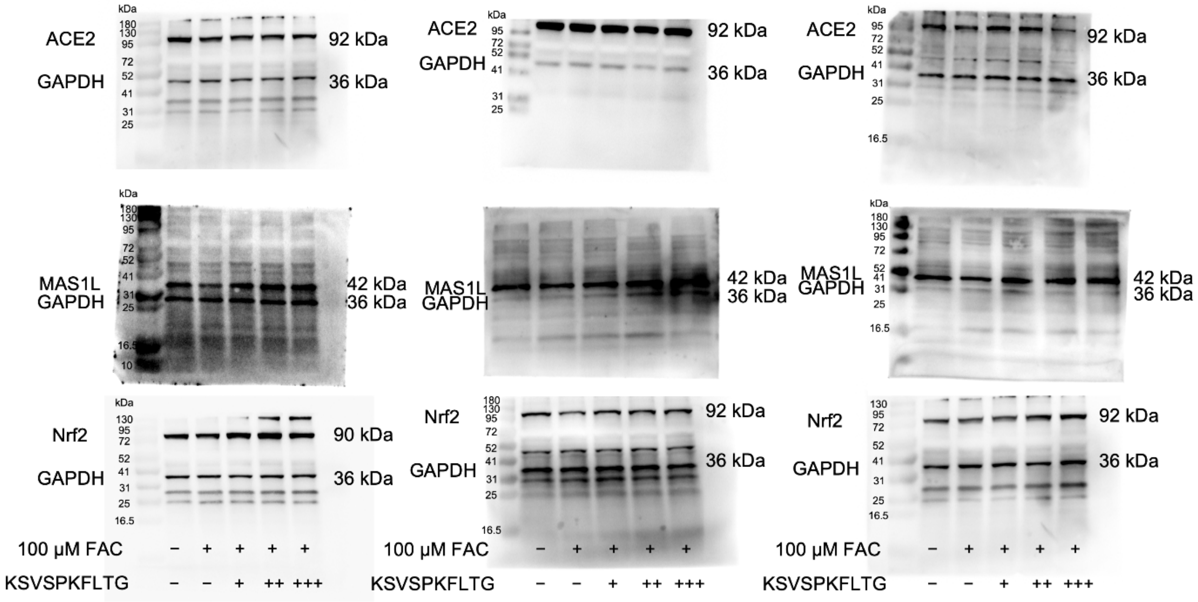

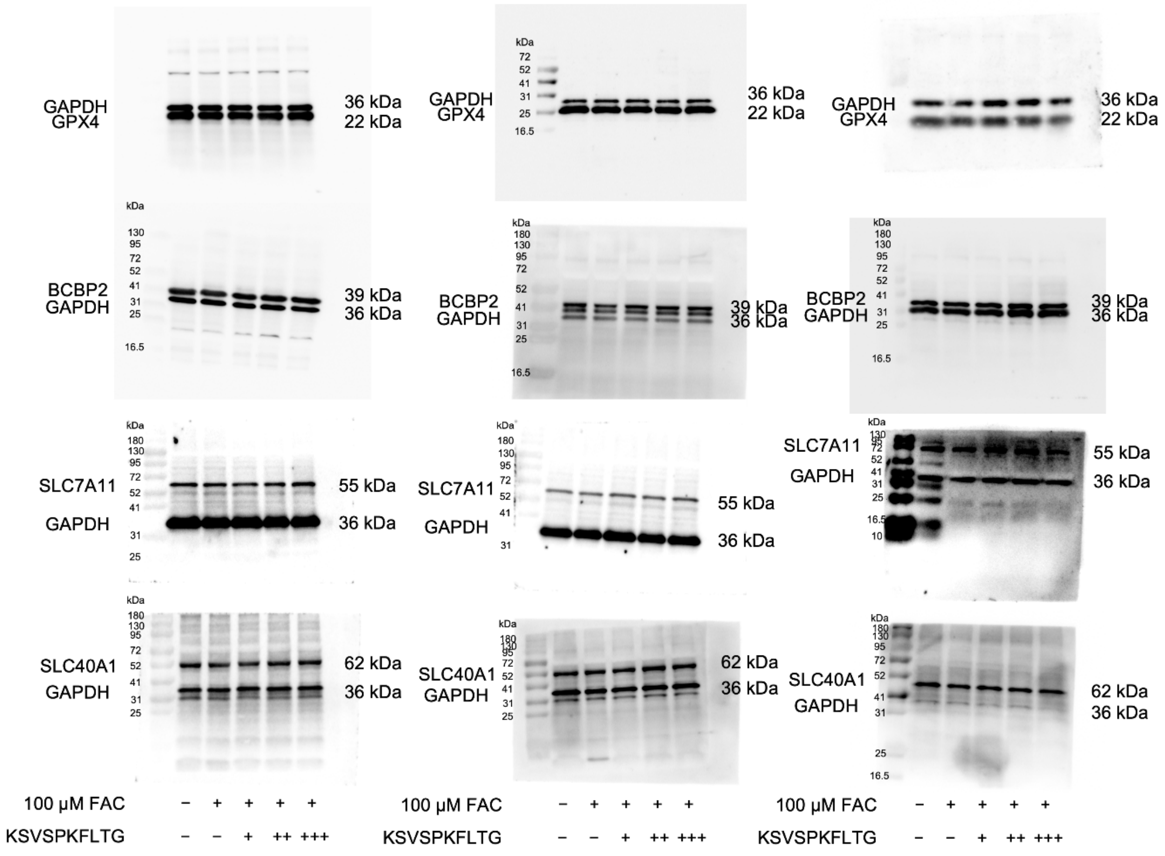

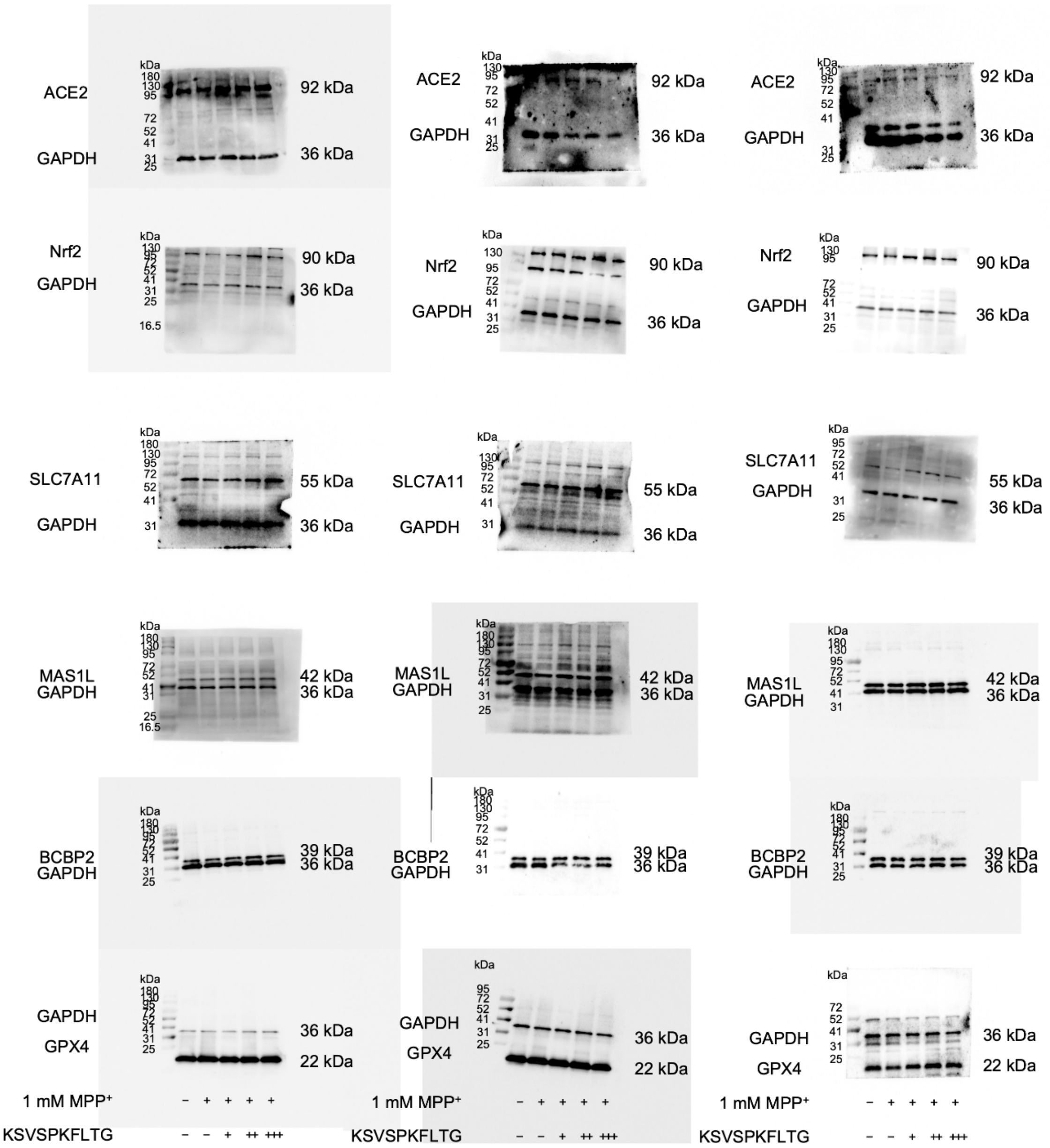
ACE2
92 kDa
GAPDH
36 kDa
Nrf2
90 kDa
GAPDH
36 kDa
SLC7A11
55 kDa
GAPDH
36 kDa
MAS1L
42 kDa
GAPDH
36 kDa
BCBP2
39 kDa
GAPDH
36 kDa
GAPDH
36 kDa
GPX4
22 kDa
1 mM MPP⁺
– + + + +
KSVSPKFLTG
– – + ++ +++
